# Suppressive effects of increasing mungbean density on growth and reproduction of junglerice and feather fingergrass

**DOI:** 10.1038/s41598-023-32320-1

**Published:** 2023-04-03

**Authors:** Amar Matloob, Ahmadreza Mobli, Bhagirath Singh Chauhan

**Affiliations:** 1 Department of Agronomy, Muhammad Nawaz Shareef University of Agriculture, Multan, Pakistan; 2 Queensland Alliance for Agriculture and Food Innovation (QAAFI), University of Queensland, Gatton, QLD 4343 Australia; 3 Department of Agronomy, University of Wisconsin-Madison, Madison, WI USA; 4 School of Agriculture and Food Sciences (SAFS), The University of Queensland, Gatton, QLD 4343 Australia; 5 Chaudhary Charan Singh Haryana Agricultural University (CCSHAU), Hisar, Haryana 125004 India

**Keywords:** Environmental sciences, Environmental impact, Agroecology, Community ecology

## Abstract

Increased planting density can provide crops a competitive advantage over weeds. This study appraised the growth and seed production of two noxious grassy weeds, i.e. feather fingergrass (*Chloris virgata* SW.) and junglerice [*Echinochloa colona* (L.) Link] in response to different mungbean [*Vigna radiata* (L.) R. Wilczek] densities (0, 82, 164, 242, and 328 plants m^−2^). A target-neighbourhood study was conducted using a completely randomized design with five replications, and there were two experimental runs in 2016–2017. The leaf, stem, and total aboveground biomass of *C. virgata* was 86, 59, and 76% greater than *E. colona*. For seed production, *E. colona* outnumbered *C. virgata* by producing 74% more seeds. Mungbean density-mediated suppression of height was more pronounced for *E. colona* compared with *C. virgata* during the first 42 days. The presence of 164–328 mungbean plants m^−2^ reduced the number of leaves of *E. colona* and *C. virgata* by 53–72% and 52–57%, respectively. The reduction in the inflorescence number caused by the highest mungbean density was higher for *C. virgata* than *E. colona*. *C. virgata* and *E. colona* growing with mungbean produced 81 and 79% fewer seeds per plant. An increase in mungbean density from 82 to 328 plants m^−2^ reduced the total aboveground biomass of *C. virgata* and *E. colona* by 45–63% and 44–67%, respectively. Increased mungbean plant density can suppress weed growth and seed production. Although increased crop density contributes to better weed management, supplemental weed control will be needed.

## Introduction

Weeds are troublesome, aggressive, and competitive botanical pests of the croplands that pose multi-dimensional problems in every cropping system, the most significant of which is the reduction in crop yields due to weed interference. On a monetary basis, weeds cause a huge loss of AU$ 4.0 billion (AU$ 1.5 billion spent on weed control measures and AU$ 2.5 billion as production losses) to Australian farmers^1,2^. Junglerice [*Echinochloa colona* (L.) Link.] is a problematic, annual, C_4_ grass weed infesting 35 cropping systems in more than 60 countries across the globe, with widespread distribution especially in Asian, African, and Australian tropical and subtropical regions^3^. In northern cropping systems of Australia, *E. colona* is an important summer annual weed^3–5^. Germination in multiple flushes, high dry matter accumulation, growth rate, profuse tillering, competitive ability, early flower bud initiation accompanied by seed output, and the inhibitory allelopathic potential make this weed noxious and troublesome^3,6–8^. Over-reliance on glyphosate as a sole means to control this weed in summer fallows has led to the evolution of herbicide-resistant biotypes in the USA, Argentina, and Australia^9^. The largest area containing glyphosate-resistant *E. colona* occurs in three Australian states, i.e., New South Wales, Queensland, and Western Australia. In Australia, *E. colona* has the second highest number of glyphosate-resistant biotypes, right after rigid ryegrass (*Lolium rigidum* Gaud.)^10^.

Feather fingergrass (*Chloris virgata* Sw.), a C_4_, summer annual weed species, is becoming increasingly problematic as it has infested large cropping areas in central Queensland in recent years and is currently invading and becoming a major problem in southern Queensland and northern New South Wales^11,12^. Being a prolific seed producer with dispersal through both wind and water, and tolerant to glyphosate, this weed has shown high adaptation to zero-till cropping systems^13,14^. Moreover, this weed also acts as a host for aphids^15^, disease-transmitting viruses^16^, and pathogenic nematodes^15^. It grows rapidly and can set seeds within 42 days^11^. Tall growing habits and a prolonged emergence period make this weed a prolific seed producer (> 140,000 seeds per plant)^17^. This species was included among the top 10 weeds in national rankings and the top four weeds of the northern grain region ranking^1^. In the field, the presence of 45–49 plants m^−2^ of *C. virgata* reduced mungbean [*Vigna radiata* (L.) R. Wilczek] seed yield by 65–73%^18^. Reductions of 20, 27, 34, and 43% in mungbean seed yield were recorded at *E. colona* infestation levels of 4, 8, 16, and 32 plants m^−2^, respectively^19^.

Glyphosate-resistant biotypes of problematic weeds are a major threat to the sustainability of reduced tillage cropping systems. Weed management has become an expensive and challenging task in the northern grain region of Australia. *Chloris virgata* and *E. colona* have become difficult-to-control weeds owing to the rise in their glyphosate resistance biotypes and are now posing serious challenges in summer fallows and crops like cotton (*Gossypium hirsutum* L.), sorghum [*Sorghum bicolor* (L.) Moench], and mungbean etc^18,20,21^. The losses in grain yield caused by *E. colona* and *C. virgata* accounted for 77,734 and 39,329 tonnes per annum, amounting to AU$ 14.7 and 7.7 million, respectively^1^. The initial cases of glyphosate resistance in *C. virgata* were reported in 2015^9^ and to date, several cases have been reported^12,22^. For *E. colona*, the first glyphosate-resistant biotype was reported in 2007, and the number of cases is increasing to date^9^. Estimates indicate that infestation of crop fields with glyphosate-resistant grasses, including *E. colona,* will increase the cost incurred in controlling weeds by AUD 40–90 ha^−1^^18^. Besides glyphosate, several Acetyl CoA Carboxylase (ACCase) inhibitors are also used for post-emergence control of annual grassy weeds. Nevertheless, resistance to these herbicides in grassy weeds has also been documented^9^.

Continuous cropping of grain cereals remains the conventional practice in Australia. Nevertheless, this form of cropping has resulted in the mining of soils for major nutrients and the stagnant or declining crop productivity coupled with reduced grain protein contents and financial returns to producers. The incorporation of pulse crops, especially mungbean, into the rotation can help overcome these issues. Mungbean is a major pulse crop grown during summer in the northern grain region and Australia exports 90% of its production to Asian countries^23^.

Chemical weed control becomes difficult when crops are infested with herbicide-resistant weeds, which could also evolve resistance to other herbicidal molecules. Sustainable weed management has become increasingly important in the backdrop of climate change, the evolution of resistant weed biotypes, and increasing food security concerns. Chemical weed control should be used in integration with different agronomic practices that affect the dynamics of crop-weed competition. In this way, sole dependence on one weed control method is minimized. Incorporating other weed management tactics (e.g., crop competition) that can supplement herbicidal weed control could be a pragmatic approach. Understanding the influence of crop management practices on the growth and reproductive behaviour of associated weeds is crucial for optimizing such practices in favour of the crop. Moreover, weeds occur in the mixture, and it is vital to ascertain the competitive effects on diverse species rather than a single weed. A substantial reduction in weed growth and reproductive output of weeds can be achieved by manipulation of the crop plants’ orientation and spacing. Reducing row spacing in mungbean to 25 cm reduced weed biomass by > 70% compared with wider spacing of 75 cm^24^. Our previous studies reported suppressive effects of increased mungbean planting densities on broad-leaved weeds like annual sowthistle (*Sonchus oleraceus* L.)^25^. It was hypothesized that increasing mungbean plant densities will negatively but differentially affect the growth of these grassy weeds. The present study was conducted to appraise whether increasing mungbean density can suppress problematic grassy weeds like *E. colona* and *C. virgata*. Another objective was to ascertain the relative competitiveness of these weeds with mungbean and to understand which weed can be easily managed by enhanced crop competition.

## Materials and methods

### Experimentation

Seeds of *E. colona* and *C. virgata* were collected from the Gatton research fields of the University of Queensland, Queensland, Australia (latitude 27.33° S, longitude 152.16° E and altitude 94 m a.s.l.) in the 2016 summer season. Plastic pots (25 cm diameter and 30 cm height) were filled with potting mix (Searles® Premium Potting Mix) and placed in a screenhouse. Three seeds of *E. colona* and *C. virgata* were sown in the center of each pot either alone (0 mungbean plant) or with 4, 8, 12, and 16 mungbean plants (cv. Jade AU; corresponding to 82, 164, 246, and 328 plants m^−2^) to quantify their response to crop interference. One healthy weed seedling was maintained per pot after thinning within 10 days after sowing (DAS). Various mungbean crop densities per pot were maintained as per treatment by sowing crop seeds equidistant from each other. The seeds were sown at a depth of 3 cm and a distance of 10 cm from weed seeds. The selected densities represent different levels of shading caused by mungbean crops after canopy closure. The weed and crop plants emerged within 6–8 DAS. The pots were irrigated daily using an automated irrigation system in such a way that moisture was not limited. The experimental pots were placed at a distance of 50 cm from each other and moved to a new position on a weekly to avoid any position effect.

### Data collection

The effect of mungbean crop interference on *E. colona* and *C. virgata* growth and seed production was quantified by measuring plant height, and number of leaves, tillers, inflorescence, and seeds. The data on plant height and number of leaves and tillers were recorded on a biweekly and continued for *E. colona* till 56 DAS and for *C. virgata* till 70 DAS. The height of weed plants was measured from the base of the plant to the tip of the uppermost leaf. For *E. colona*, the study was terminated at 56 DAS when lower leaves of this weed started to senesce. *Chloris virgata* was harvested at 70 DAS (as its plants grew for additional two weeks compared with *E. colona*) when its leaves became yellowish. At harvest, the number of inflorescences and seeds plant^−1^ for *E. colona* and *C. virgata* were counted. The aboveground biomass of these weeds was measured after drying the harvested plant samples in an oven at 70 C for 72 h. Height and aboveground biomass of mungbean were measured at harvest. During these studies, no insect attack or disease incidence was observed, and hence no curative measures were undertaken.

### Experimental design and data analyses

This study was conducted using a completely randomized design with five replications, and there were two experimental runs from September 2016 to May 2017. The next experimental run was initiated within a month of the termination of the previous run. Before analyses, the homogeneity and normality of the data were checked and analysis of variance (ANOVA) was performed using GenStat (18th edition; VSN International, Hemel Hempstead, UK). Data were pooled across the runs (a total of 10 replications) for further statistical analyses as no significant interaction between treatments and experimental runs were observed. Differences amongst treatment means were evaluated by Fisher’s protected least significant differences (LSD, *p* ≤ 0.05) test.

A three-parameter sigmoid model (Eq. 1) was fitted to the data pertaining to height, the number of leaves, and tillers plant^−1^ of *E. colona* and *C. virgata*:

1 $$\mathrm{Y}=\frac{a}{1+{\mathrm{e}}^{-\left(\frac{{\mathrm{X}-\mathrm{T}}_{50}}{\mathrm{b}}\right)}}$$

Here *Y* is the predicted height, number of leaves, or tillers plant^−1^, *a* is the maximum predicted height, number of leaves, or tillers plant^−1^, *T*_50_ is the time needed for achieving 50% of maximum predicted height, number of leaves, or tillers plant^−1^, and *b* was the rate of height, and number of leaves, or tillers plant^−1^ increment (slope). Nonlinear regression was performed by Sigmaplot software (version 14; Systat Software, Inc, San Jose, CA, USA), and predicted values were compared using the standard error of means.

The effect of increasing density of mungbean on the reduction of *E. colona* and *C. virgata* biomass was modeled using a two-parameter exponential decay curve (Eq. 2):

2 $$\mathrm{Y}= {ae}^{-bx}$$

Here *Y* is predicted biomass, *a* is a constant parameter, and *b* is the rate of biomass reduction (Slope). The fitness of the fitted models was ascertained in terms of *R*^2^ values.

## Results

### Plant height

**Figure 1:**
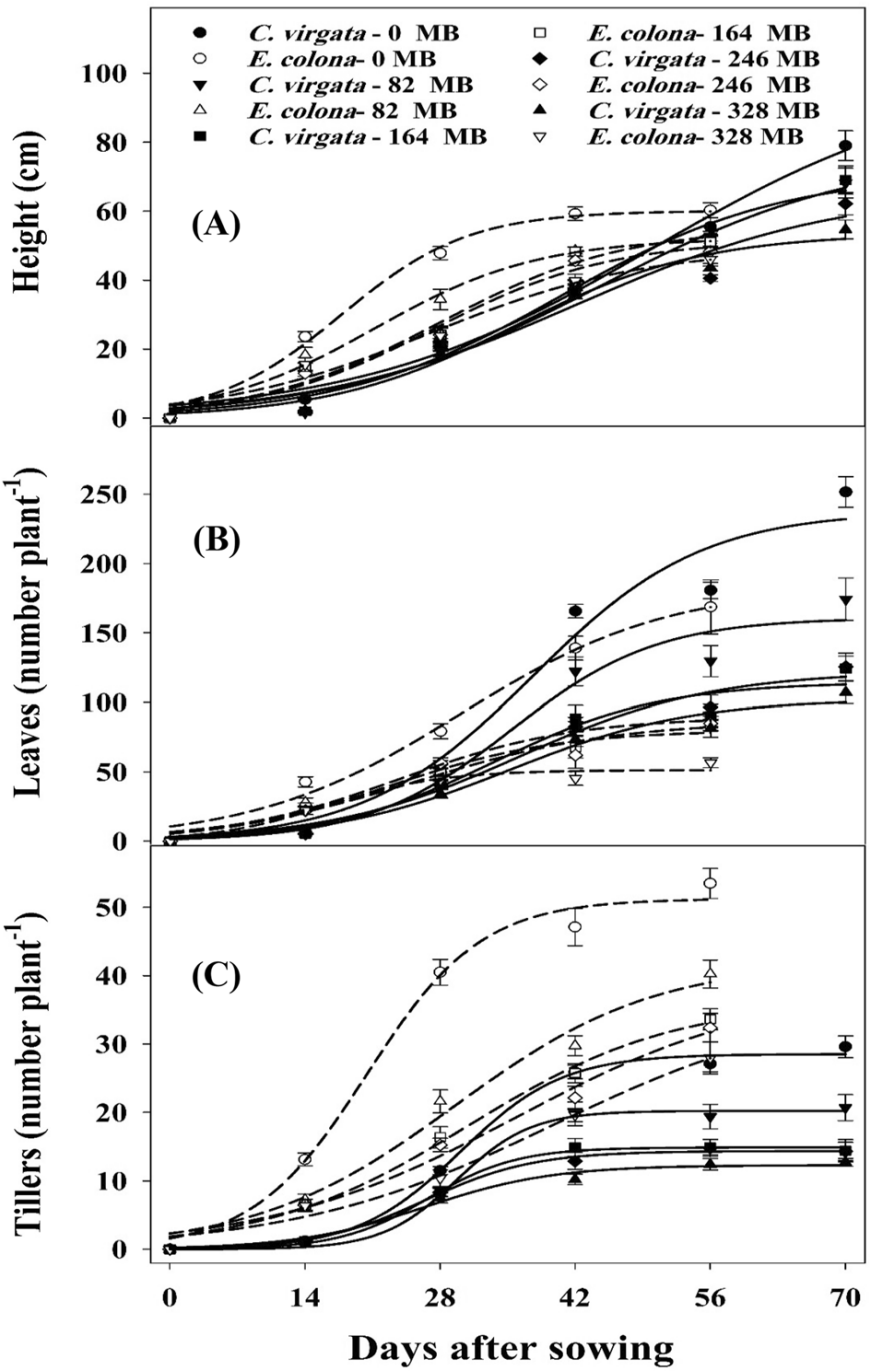
(**A**) Plant height (cm), (**B**) tiller number per plant, and (**C**) leaf number per plant of C. virgata and E. colona when grown alone (0 MB) or in competition with 82, 164, 246 and 328 mungbean (MB) plants. The lines represent a three-parameter sigmoid model (y = a/{1 + exp[− (X − T50)/b]}) fit to plant height, leaf number per plant, and tiller number per plant. The capped bars represent the standard error of the mean. Parameter estimates of the model are given in Table 1.

The plant height of *E. colona* and *C. virgata* manifested a sigmoidal response (Fig. 1A). Both weeds growing alone, without mungbean interference, recorded maximum plant height. Plants of *E. colona* were taller than *C. virgata* at 42 DAS. Afterward, *C. virgata* recorded greater height, and its final plant height was 40% greater than *E. colona*. During the first 42 DAS, differences for mungbean density-related suppression of plant height were more pronounced for *E. colona* compared with *C. virgata*. The presence of 82 mungbean plants m^−2^ significantly suppressed the plant height of *E. colona* more than the control (0 mungbean plant m^−2^). A further increase in mungbean density had a greater suppressive effect, yet 164, 246, and 328 mungbean plants m^−2^ resulted in similar suppression of *E. colona* plant height. However, these aforementioned mungbean densities had a similar effect on the plant height of *C. virgata* till 42 DAS (Fig. 1A). However, higher mungbean densities (246 and 328 plants m^−2^) caused significant suppression in plant height of *C. virgata* at 70 DAS as compared with 0, 82, and 164 mungbean plants m^−2^. A three-parameter sigmoidal model estimated a maximum plant height of 60 and 100 cm for *E. colona* and *C. virgata*, respectively. Regression estimates showed that increasing mungbean density from 82 to 328 plants m^−2^ reduced the height of *E. colona* and *C. virgata* by 8–18% and 23–46%, respectively, as compared with their plants grown alone, indicating greater height suppression of *C. virgata* weed species in response to mungbean interference (Table 1). The time required to attain 50% height by *E. colona* and *C. virgata* plants grown alone was 18 and 50 days*,* respectively. In the presence of 328 mungbean plants, this time was reduced by 29% for *C. virgata*. Under the same treatment, *E. colona* took 27 days to achieve 50% height as against 18 days, when it grew without mungbean interference (Table 1).

**Table 1:** Estimated parameters (± SEs) of a three-sigmoid model, f = a/(1 + exp(− (*X* − *T*_50_)/b)) , fit to height, number of leaves and tillers of *C. virgata* and *E. colona* in competition with different densities (0, 82, 164, 246, and 328 plants m^−2^) of mungbean.

Mungbean (MB) density (plant m^−2^)	Parameters (± SEs)			
	a	b	X0	R^2^
Plant height (cm)				
*C. virgate*—0 MB	99.97 ± 18.84	15.35 ± 3.39	50.15 ± 7.34	0.99
*E. colona*—0 MB	60.08 ± 2.73	6.58 ± 1.35	17.79 ± 1.60	0.99
*C. virgata*—82 MB	71.05 ± 7.78	11.42 ± 2.74	40.59 ± 3.99	0.98
*E. colona*—82 MB	52.19 ± 3.75	8.43 ± 2.08	21.25 ± 2.56	0.99
*C. virgata*—164 MB	77.04 ± 14.45	13.47 ± 4.05	44.56 ± 7.07	0.98
*E. colona*—164 MB	55.39 ± 7.68	9.53 ± 3.40	27.94 ± 4.55	0.97
*C. virgata*—246 MB	66.13 ± 17.18	13.67 ± 6.11	42.27 ± 10.18	0.95
*E. colona*—246 MB	51.74 ± 5.77	9.17 ± 2.79	27.30 ± 3.65	0.98
*C. virgata*—328 MB	53.52 ± 4.46	9.73 ± 2.38	35.78 ± 3.08	0.98
*E. colona*—328 MB	49.34 ± 7.48	11.10 ± 3.84	26.96 ± 5.37	0.97
Leaves (number plant^−1^)				
*C. virgata*—0 MB	237.77 ± 28.51	8.75 ± 3.27	37.56 ± 4.15	0.97
*E. colona*—0 MB	184.07 ± 20.86	10.81 ± 2.57	30.13 ± 3.80	0.99
*C. virgata*—82 MB	160.69 ± 15.44	7.52 ± 2.66	35.36 ± 3.34	0.97
*E. colona*—82 MB	89.58 ± 7.78	9.07 ± 2.39	23.69 ± 3.02	0.98
*C. virgata*—164 MB	114.49 ± 12.68	8.67 ± 3.29	33.57 ± 4.10	0.96
*E. colona*—164 MB	86.54 ± 9.83	10.30 ± 3.01	25.51 ± 3.99	0.98
*C. virgata*—246 MB	112.25 ± 11.64	9.95 ± 2.70	36.19 ± 3.52	0.98
*E. colona*—246 MB	80.02 ± 11.28	8.73 ± 3.96	22.72 ± 4.93	0.96
*C. virgata*—328 MB	102.47 ± 9.78	9.42 ± 2.77	35.09 ± 3.53	0.98
*E. colona*—328 MB	51.25 ± 4.50	5.91 ± 2.82	16.17 ± 3.00	0.97
Tillers (number plant^−1^)				
*C. virgata*—0 MB	28.52 ± 0.70	5.23 ± 0.81	30.10 ± 0.81	0.99
*E. colona*—0 MB	51.24 ± 2.34	5.93 ± 1.15	20.43 ± 1.55	0.99
*C. virgata*—82 MB	20.23 ± 0.51	3.85 ± 1.09	29.71 ± 0.80	0.99
*E. colona*—82 MB	42.05 ± 5.66	10.43 ± 3.11	29.58 ± 4.46	0.98
*C. virgata*—164 MB	14.89 ± 0.30	4.59 ± 0.78	26.76 ± 0.62	0.99
*E. colona*—164 MB	36.27 ± 3.47	10.61 ± 2.10	30.99 ± 3.13	0.99
*C. virgata*—246 MB	14.35 ± 0.35	5.60 ± 0.85	26.45 ± 0.84	0.99
*E. colona*—246 MB	38.61 ± 8.87	13.06 ± 4.16	35.90 ± 7.94	0.98
*C. virgata*—328 MB	12.29 ± 0.59	6.64 ± 1.64	26.35 ± 1.81	0.99
*E. colona*—328 MB	36.43 ± 9.25	13.61 ± 3.90	39.78 ± 8.60	0.89

*a* is the maximum predicted height, number of leaves, or tillers plant^−1^, *T*_50_ is the time needed for achieving 50% of maximum predicted height, number of leaves, or tillers plant^−1^, *b* was the rate of height, and number of leaves, or tillers plant^−1^ increment (slope), and R^2^ is coefficient of determination.

### Number of leaves

The number of leaves of both species showed a temporal increase (Fig. 1B). Leaves were more numerous for *C. virgata* plants growing alone than *E. colona.* The increasing mungbean density had a negligible effect on the number of leaves of both weeds till 14 DAS. However, afterward, differences among the treatments became obvious, and increasing mungbean densities significantly reduced the number of leaves of *E. colona* and *C. virgata* at 56 DAS*.* Increasing mungbean density from 0 to 82 plants m^−2^ reduced the number of leaves of *C. virgata* from 238 to 161 per plant, corresponding to a 32% reduction (Table 1). For *E. colona,* the reduction at the same crop density was 51%. Compared with the number of leaves of *E. colona* and *C. virgata* that grew without mungbean interference, the presence of 164–328 mungbean plants m^−2^ reduced this parameter by 53–72% and 52–57%, respectively (Table 1). The lowest number of leaves for both weeds was noted when these were grown in competition with 328 mungbean plants m^−2^. The prediction of the fitted model for 50% leaf production by *C. virgata* was similar for all the tested mungbean densities. However, for *E. colona*, the regression model indicated that the time required to produce 50% leaves was curtailed by 46% in the presence of 328 mungbean plants m^−2^ compared with no mungbean interference.

### Number of tillers

In the absence of any interference, *E. colona* plants produced a higher (83%) number of tillers than *C. virgata.* The increasing densities of mungbean plants m^−2^ suppressed the tillering ability of both weeds (Fig. 1C). The tillering of *E. colona* and *C. virgata* was reduced by 18 and 29% when these weeds were grown in competition with 82 mungbean plants m^−2^. A further increase in mungbean density to 164 plants m^−2^ increased the suppression to 29 and 48%, respectively. The presence of 246 and 328 mungbean plants m^−2^ caused a similar reduction in tillering of both weeds; although the overall reduction was much greater for *C. virgata* than *E. colona* at all the tested mungbean densities. The slope (*b*) increased for *E. colona* with an increase in the mungbean density (Table 1). Over the increasing mungbean densities (164–328 plants m^−2^), the time required to achieve 50% of the maximum predicted tillers remained similar for *C*. *virgata* (Table 1). However, for *E. colona*, it was longer by 9 days, when density was doubled from 164 to 328 plants m^−2^. For the time required to achieve 50% of the maximum predicted tillers under increased mungbean density, a decreasing trend was observed for *C. virgata,* while the opposite was true for *E. colona*.

### Inflorescence and seed production

**Figure 2:**
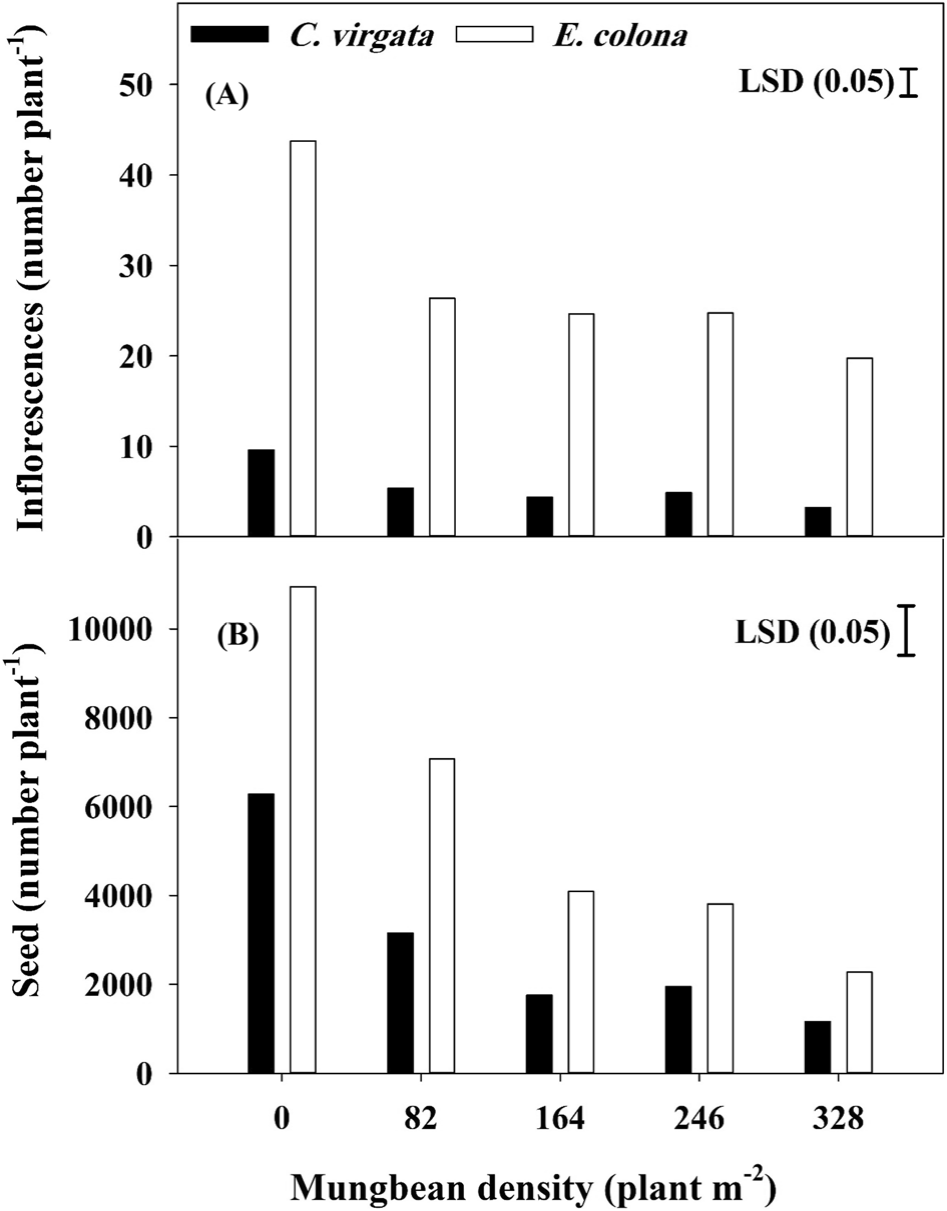
(**A**) Inflorescence number per plant and (**B**) Seed production per plant of *C. virgata* and *E. colona* when grown alone (0 MB) or in competition with 82, 164, 246 and 328 mungbean (MB) plants. Capped bar denotes LSD values at *p* ≤ 0.05.

The increasing mungbean densities significantly suppressed the inflorescence number per plant of both weed species. However, species-specific differences were observed in response to the applied treatments. Regardless of crop interference, the inflorescences per plant were more numerous in the case of *E. colona* than *C. virgata* (Fig. 2A). When grown without mungbean interference, *E. colona* produced 44 inflorescences per plant as compared to only 10 inflorescences produced by *C. virgata*. Increasing the mungbean density from 82 to 328 plants m^−2^ caused reductions ranging from 46 to 68% for *C. virgata* plants. The reduction in the inflorescence number of *E. colona* due to the presence of 82–246 mungbean plants m^−2^ ranged from 40 to 44%. However, a further increase in density beyond 246 plants m^−2^ increased the suppression magnitude by 55% (Fig. 2A). The reduction in the inflorescence number due to interference posed by the highest mungbean density (328 plants m^−2^) was higher for *C. virgata* than *E. colona,* when compared with their respective controls.

Regarding seed production, *E. colona* outnumbered *C. virgata* and produced 74% more seeds (Fig. 2B). Increasing the mungbean density from 82 to 164 plants m^−2^ reduced the number of seeds of *C. virgata* from 3155 to 1759 per plant corresponding to a 44% reduction (Table 1). For *E. colona,* the corresponding reduction was 42%. The presence of 164 and 246 mungbean plants m^−2^ caused a similar reduction in the seed output for *E. colona* as well as *C. virgata.* The aforementioned mungbean densities caused differential suppression of the tested weeds (Fig. 2B). A further increase in mungbean density to 328 plants m^−2^ caused a significant reduction (79% compared with the respective control) in the number of seeds produced by *E. colona*, whereas, for *C. virgata*, the reduction in the seed output at this mungbean density was similar to that achieved at 164 and 246 mungbean plants m^−2^.

### Weed biomass

**Figure 3:**
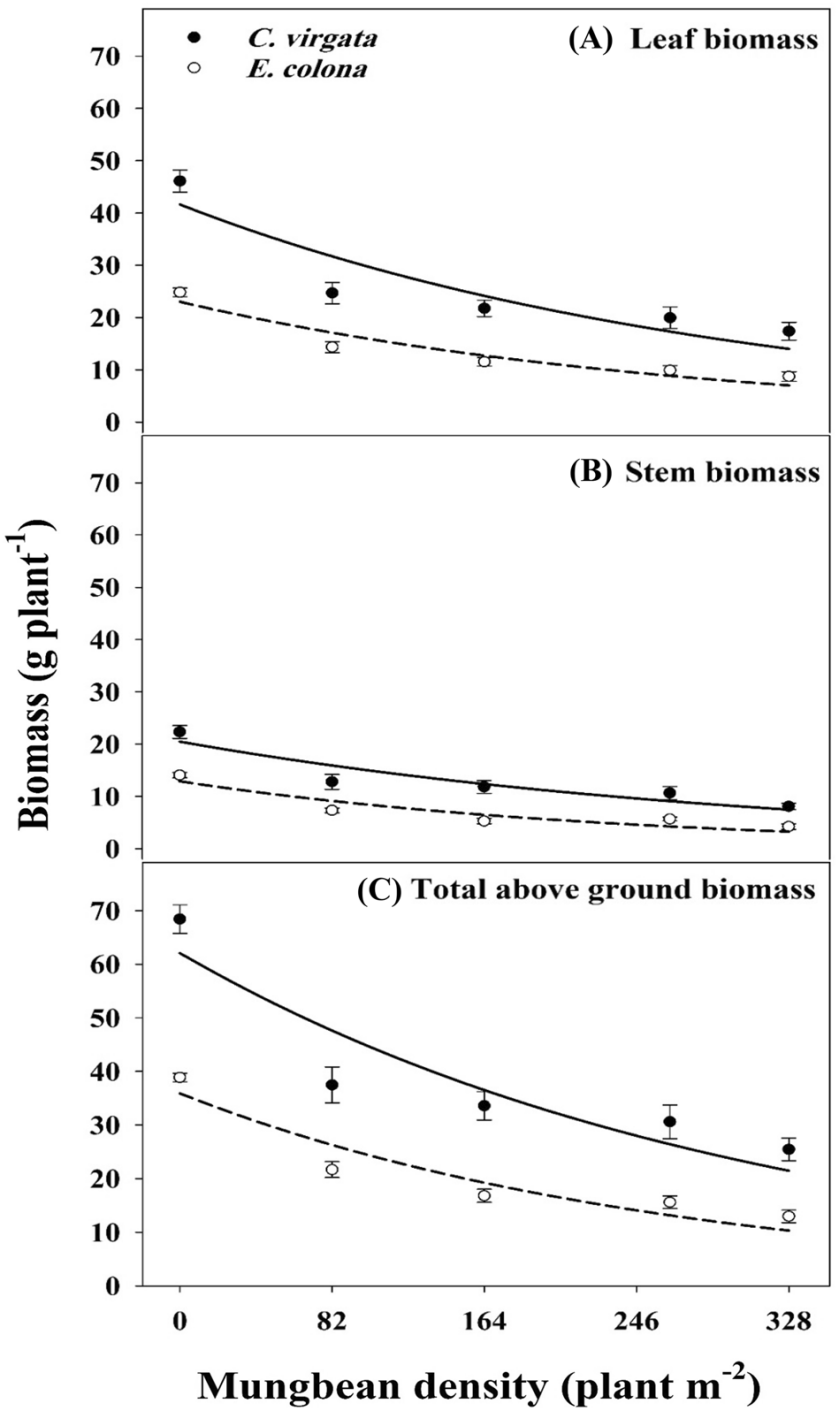
(**A**) Stem (**B**) leaf and (**C**) total aboveground biomass of *C. virgata* and *E. colona* when grown alone (0 MB) or in competition with 82, 164, 246 and 328 mungbean (MB) plants. The lines represent a two-parameter exponential decay model, Y = ae^−bx^, fit to leaf, stem, and total aboveground biomass per plant (g plant^−1^). The capped bars represent the standard error of the mean. Parameter estimates of the model are given in Table 2.

Increasing mungbean densities suppressed the leaf, stem, and total aboveground biomass of both weed species (Fig. 3A–C; Table 2). In general, biomass accumulation by *C. virgata* was greater than *E. colona*. The highest biomass by both weeds was produced under no interference conditions. In the absence of any mungbean plant, the leaf, stem, and total aboveground biomass produced by *C. virgata* were 86, 59, and 76% greater than *E. colona*. Increasing mungbean density from 0 to 82, 164, 246, and 328 plants m^−2^ decreased leaf biomass of *C. virgata* by 46, 53, 57, and 62%, respectively. The corresponding reductions in the leaf biomass of *E. colona* were 42, 54, 60, and 65%, respectively (Fig. 3A). Stem biomass was less than leaf biomass for both weed species. In response to an increase in mungbean density from 82 to 328 plants m^−2^, the reduction in stem biomass was 43–64% and 48–60% for *C. virgata* and *E. colona,* respectively (Fig. 3B). The reduction in total aboveground biomass of *C. virgata* and *E. colona* corresponded to 45–63% and 44–67%*,* respectively (Fig. 3C).

**Table 2:** Estimated parameters (± SEs) of a two-parameter exponential decay model, Y = ae^−bx^, fit to leaf, stem, and total shoot biomass per plant (g plant^−1^) of *C. virgata* and *E. colona* in competition with different densities (0, 82, 164, 246, and 328 plants m^−2^) of mungbean.

Weed species and plant part	Parameters (± SEs)		
	a	b	R^2^
*C. virgate*—leaf	41.62 ± 4.93	0.0033 ± 0.0009	0.89
*E. colona*—leaf	23.02 ± 2.07	0.0036 ± 0.0007	0.90
*C. virgate*—stem	20.48 ± 2.05	0.0031 ± 0.0007	0.91
*E. colona*—stem	12.90 ± 1.58	0.0042 ± 0.0011	0.89
*C. virgata*—total	62.10 ± 6.93	0.0032 ± 0.0008	0.89
*E. colona*—total	35.89 ± 3.62	0.0038 ± 0.0008	0.89

*a* is a constant parameter, *b* is the rate of biomass reduction (slope), and *R*^2^ is the coefficient of determination.

### Mungbean plant height and biomass

The height of mungbean plants remained unaffected by their increasing densities (Table 3). However, the density-mediated difference for mungbean biomass was significant. Increasing densities increased mungbean biomass and such an increase was pronounced for mungbean plants growing in association with *C. virgata* than *E. colona*. Mungbean densities of 82, 164, 246, and 328 plants m^−2^ recorded 19, 11, 21, and 26% less biomass when mungbean plants at these densities grew with *E. colona* compared with *C. virgata*. The mungbean biomass manifested an increase of 11, 22, and 35% when its density (growing in association with *C. virgata*) was increased from 82 plants m^−2^ to 128, 246, and 328 plants m^−2^. The increase in mungbean biomass at aforementioned mungbean densities growing in competition with *E. colona* corresponded to 20, 18, and 23%, respectively.

**Table 3:** Effect of *C. virgata* and *E. colona* competition with mungbean on mungbean height and aboveground biomass.

Mungbean (Plant m^−2^)	Weed species	
	*C. virgata*	*E. colona*
Aboveground biomass (g plant^−1^)		
82	57.2	46.6
164	63.3	56.1
246	69.7	55.1
328	77.5	57.5
LSD (0.05)	8.76	
Height (cm)		
82	35.715	34.8725
164	36.015	35.2875
246	36.395	34.9175
328	37.2675	35.2125
LSD (0.05)	NS^a^	

^a^NS: non-significant.

## Discussion

Plant height reflects the vegetative growth behaviour in response to environmental inputs during the growth period, and it has a profound influence on weed-crop competition^26^. Increasing mungbean densities had a negative effect on the plant height of both weeds. The reduction in plant height of the tested weed species could be attributed to more shading and early canopy closure at higher mungbean densities. The resource (light, space, nutrient, and water) deprivation can be responsible for such observed effects. Although increased crop competitiveness through the increased number of mungbean plants suppressed the height of *E. colona* and *C. virgata*, the height of both weed species was greater than the mungbean plants at harvest.

A recent study also reported that increasing crop densities alone cannot completely suppress the height of problematic weeds like *S. oleraceus*^25^. For increased crop competition to be effective, the crop species should grow taller than the weeds and produce profuse branches/tillers^27,28^. The ability of these grassy weeds to grow taller than mungbean plants, even at higher crop densities, suggests their capacity to overcome crop interference by shade-avoiding characteristics. Moreover, the plant height of both weeds differed on a temporal scale with *E. colona* growing taller than *C. virgata* for the first 42 days, with *C. virgata* achieving the maximum final height later in the season. This could be attributed to the divergence in growth habits and the life cycle of these grassy weeds. A previous study revealed that the effect of increasing crop competition on the height of weed plants is species-specific^29^. For example, increasing rice (*Oryza sativa* L.) density averted the height of longfruited primrose-willow [*Ludwigia octovalvis* (Jacq.) P. H. Raven] by 41%, while it did not affect the height of spiny amaranth (*Amaranthus spinosus* L.). The difference in plant height of the tested weeds may be due to genetic differences; however, their expression in the present study was also modified to a great extent by the different treatments imposed. Variations in plant height of *C. virgata* and *E. colona* could be attributed to variable effects of crop competition (light and nutrients) offered by different mungbean densities under different treatments.

The reduction in the number of leaves of target weeds in response to increased mungbean density suggests the effectiveness of crop interference in reducing weed growth. Leaf, being an assimilatory surface, is an important plant organ, especially for light interception and competition. With an increase in planting density of corn (*Zea mays* L.), the ability of weeds to capture light was decreased^30^ presumably because of reduced light transmittance and increased corn leaf area. Another study documented a 61–85% reduction in the number of leaves of *E. colona*, when this weed was grown in association with rice plants as compared to plants of this weed grown alone^31^. After 42 DAS, weeds, especially *C. virgata*, were able to grow taller than the mungbean and thus availed an opportunity to produce a greater number of leaves afterward.

Niche pre-emption has been proposed as a prime mechanism conducive to suppression in the growth and development of weeds caused by increased crop densities^32,33^. Crops like mungbean, when grown at high planting densities, could utilize the space and growth resources that otherwise would be taken by weed plants. In the absence of mungbean interference, weeds produced abundant tillers owing to the availability of ample light, space, and nutrients. Phenotypic plasticity allows the weeds to adjust their growth behavior in response to prevailing agroclimatic conditions^34^. Mungbean plants, because of their spreading growth habit and quick canopy closure, especially at high plant densities, might have reduced light transmittance to the weed plants besides causing the smothering effect, which possibly explains the reduction in tillering of both weed species. The results suggest that increasing mungbean densities suppressed the tillering of *C. virgata* more effectively than *E. colona,* since tillering was more profuse in the case of *E. colona*.

The reduction in the inflorescence number under high mungbean densities suggests that mungbean can reduce reproductive fitness of the tested weeds compared to when these weeds grew alone. Thus, a reduction in the seed output and hence population density can be expected in the next season. Depletion of growth resources and diminished vegetative growth of the tested weeds in the presence of mungbean plants could explain fewer inflorescences produced by *E. colona* and *C. virgata*. Negative implications of increased crop interference on related weed species are documented elsewhere. The inflorescence biomass of barnyardgrass (*Echinochloa crus-galli* (L.) P. Beauv) was four times higher in the absence of rice interference^34^. A recent study reported a 74–91% reduction in the number of inflorescences of *E. colona* in response to increased mungbean interference^28^ which was higher compared with that recorded in the present study. However, glyphosate-resistant and -susceptible biotypes used in the study of Mutti et al.^28^ were similar to each other for their inflorescence number and seed output, indicating that there is no fitness penalty associated with glyphosate resistance in *E. colona*.

.Finding of this research suggests that any further increase in mungbean density beyond 164 plants m^−2^ had a non-significant effect on the reproductive success of *C. virgata*. Seed production is an important attribute governing the weed seedbank and overall weed population dynamics under field conditions^21^. Our results suggest that interference by a dense crop stand can help in reducing *C. virgata* and *E. colona* seed production and support the recommendation of a fast-growing, weed-suppressive, and competitive crop stand to suppress weeds. With an increase in mungbean densities, a reduction in vegetative growth attributes like height, number of leaves and tillers, and biomass (discussed in the next section) of target weeds was observed, which in turn was conducive to reduced seed output.

Although *C. virgata* and *E. colona* growing in association with mungbean produced 81 and 79% fewer seeds per plant, this reduced seed output is capable enough to cause heavy infestations in the next growing seasons^34,35^. At the highest mungbean density (328 plants m^−2^), *C. virgata* and *E. colona* were still able to produce 1164 and 2271 seeds, respectively. Nevertheless, compared to the enormous seed fecundity of these weeds (when grown alone), the use of increased mungbean densities seems promising to reduce contribution to the seedbank, but at the same time warrants the need for additional control measures. Hence, it can be inferred that to manage weeds that are prolific seed producers, as is the case with *C. virgata* and *E. colona*, sole reliance on increased crop densities to suppress weeds is not viable for long-term weed management. *E. colona* completed its life cycle within 56 days, but *C. virgata* continued to grow for another 14 days and produced seeds at a height greater than the mungbean and thus can be a potential target for harvest weed seed control in summer crops^21^. A recent study reported > 90% seed retention for *C. virgata* at mungbean harvest with very low seed dispersal^18^ suggesting the possibility for harvest weed seed control of this weed.

The reduction in seedling morphological attributes was translated into lower *E. colona* and *E. colona* plant biomass, owing to the reduction in height, number of leaves, and tillering of these weed species. The reduction in weed biomass with an increase in mungbean density is in line with the previous work of Chauhan et al.^24^ in the absence of mungbean interference, weed plants utilized the available resources to their full benefit and manifested tall plants with profuse tillering and numerous leaves. These observations suggest that during fallow periods, these weeds could be devastating due to their aggressive growth behaviour. Owing to their C_4_ mode of carbon fixation, robust growth rates, and great light-use efficiencies, these weeds can produce substantial biomass. The greater leaf and total aboveground biomass of *C. virgta* compared with *E. colona* could be in part to its extended growth period and more number of leaves. At all the mungbean densities, the leaf biomass was greater than the stem biomass for both weed species. Increased biomass allocation to leaves has been postulated as a shade-avoiding mechanism in weeds^35^. Phenotypic plasticity in weeds has been proposed as a plausible explanation to cope with the acquisition of limiting growth resources^29,33^. More biomass allocation to leaves has been reported as a shade avoidance mechanism to avoid the negative effects of shade (reduced light transmittance) caused by dense crop stands^34^. The success of weeds in competitive crop stands will not only depend on their ability to grow taller than the crop but also alter their morphological attributes and biomass partitioning^29^.

Manipulation of cultural practices can have a profound effect on weed dynamics in field crops. Planting density is an effective tool that can avert the negative effects of weed competition^36,37^. Increased planting density can cause weed suppression, besides compensating for poor crop stand establishment. Under field conditions, higher than recommended seeding rates and planting densities can be used to compensate for unforeseen biotic and abiotic stresses^38^. Higher seeding rates often impart a competitive advantage to the crop under weedy conditions, which is lost under weed-free conditions^32^. In Australia, typical mungbean planting densities are approximately 30–40 plants m^−2^. The objective of our study was to evaluate the effect of increased mungbean planting density on the suppression of grassy weeds in terms of their growth and reproductive potential. We do not recommend planting densities based on the findings of these studies. Under field conditions, the dense crop stand due to increased densities may interfere with other practices such as mechanical weed control and harvesting operations. Thus, threshold levels of crop densities need to be optimized. Due to the early maturation of the studied weeds in the present study, mungbean grain yield was not evaluated. However, this aspect should be considered in future studies to better understand the effect of weed competition.

## Data Availability

The datasets used and/or analysed during the current study available from the corresponding author on reasonable request.
